# Implementation and use of computerised clinical decision support (CCDS) in emergency pre-hospital care: a qualitative study of paramedic views and experience using Strong Structuration Theory

**DOI:** 10.1186/s13012-018-0786-x

**Published:** 2018-07-04

**Authors:** Alison Porter, Jeremy Dale, Theresa Foster, Pip Logan, Bridget Wells, Helen Snooks

**Affiliations:** 1 Patient and Population Health Research, Swansea University Medical School, Swansea, SA2 8PP UK; 2 University of Warwick, Coventry, UK; 3 East of England Ambulance Service Trust, Barton Mills, UK; 4 University of Nottingham, Nottingham, UK

**Keywords:** Paramedic, Emergency, Pre-hospital, Information technology, Decision support, CCDS

## Abstract

**Background:**

Computerised clinical decision support (CCDS) has been shown to improve processes of care in some healthcare settings, but there is little evidence related to its use or effects in pre-hospital emergency care. CCDS in this setting aligns with policies to increase IT use in ambulance care, enhance paramedic decision-making skills, reduce avoidable emergency department attendances and improve quality of care and patient experience. This qualitative study was conducted alongside a cluster randomised trial in two ambulance services of the costs and effects of web-based CCDS system designed to support paramedic decision-making in the care of older people following a fall. Paramedics were trained to enter observations and history for relevant patients on a tablet, and the CCDS then generated a recommended course of action which could be logged. Our aim was to describe paramedics’ experience of the CCDS intervention and to identify factors affecting its implementation and use.

**Methods:**

We invited all paramedics who had been randomly allocated to the intervention arm of the trial to participate in interviews or focus groups. The study was underpinned by Strong Structuration Theory, a theoretical model for studying innovation based on the relationship between what people do and their context. We used the Framework approach to data analysis.

**Results:**

Twenty out of 22 paramedics agreed to participate. We developed a model of paramedic experience of CCDS with three domains: context, adoption and use, and outcomes. Aspects of context which had an impact included organisational culture and perceived support for non-conveyance decisions. Experience of adoption and use of the CCDS varied between individual paramedics, with some using it with all eligible patients, some only with patients they thought were ‘suitable’ and some never using it. A range of outcomes were reported, some of which were different from the intended role of the technology in decision support.

**Conclusion:**

Implementation of new technology such as CCDS is not a one-off event, but an ongoing process, which requires support at the organisational level to be effective.

**Trial registration:**

ISRCTN Registry 10538608. Registered 1 May 2007. Retrospectively registered.

**Electronic supplementary material:**

The online version of this article (10.1186/s13012-018-0786-x) contains supplementary material, which is available to authorized users.

## Background

Increasing demand for healthcare in the UK has led to the development of policy and practice to reduce unnecessary hospital attendances and admissions and to promote a shift towards community-based care [1–3]. Referral pathways have already been introduced to enable paramedics to refer suitable patients to community-based care rather than convey them to emergency departments (ED). This shift reflects policies that support developing the role of paramedics as clinical decision-makers [4].

At the same time, in the UK and internationally, there is policy commitment to increasing the use of technology in healthcare [5, 6]. However, it is known that the diffusion of technology innovations in healthcare is not predictable and often poses a challenge for healthcare providers [7]. Innovations can meet resistance from clinicians, or be abandoned in the face of practical challenges, while any benefits maybe disproportionate to cost [8]. This means that, when any technological intervention is being introduced or evaluated, it is worth examining the processes of implementation and adoption into use to understand both how it might affect clinical practice and also what may inhibit change.

Computerised clinical decision support (CCDS) is one technology which has been shown to improve processes of care and, to a lesser extent, patient outcomes in other healthcare settings [9–13] by providing clinicians with algorithmic diagnostic and treatment recommendations based on patient information. Evaluations of CCDS use by physicians and nurses in an emergency context have identified implementation challenges including practical problems with the technology [14, 15] and low rates of adoption by clinicians [16–18]. CCDS has the potential to assist paramedics, but paramedics’ experience of using CCDS in the emergency pre-hospital care setting has not previously been evaluated in the published literature.

We present results of analysis of qualitative data collected from paramedics about their experience and views of CCDS, implemented within a cluster randomised trial to test effects of CCDS in the care of older people following a fall (SAFER 1) [19], conducted in two different ambulance services in the UK. The CCDS described in this paper is assessment software, based on content accredited by the National Institute for Health and Care Excellence, which was loaded onto the electronic patient record handheld devices kept by the ambulance crews. The key trial findings (Table 1) were that paramedics who had access to CCDS were twice as likely to refer patients to community-based care as paramedics without it (42/436 compared to 17/343; odds ratio 2.04, 95% CI 1.12 to 3.72). However, paramedics used CCDS with only 12.4% of eligible patients (54/436), with usage levels very different between the two study sites.

**Table 1:** Key findings from the SAFER 1 trial

Aim: To evaluate effectiveness, safety and cost-effectiveness of computerised clinical decision support (CCDS) for paramedics attending older people who fall.	
Design: A cluster randomised trial with paramedics as the unit of randomisation.	
Results:	
• 17 intervention paramedics used CCDS for 54 (12.4%) of 436 participants. CCDS usage was much lower in site 1, where CCDS and electronic data capture were both new (5/235 participants = 2%), than in site 2, where electronic data capture was already in place (49/201 participants = 24%).	
• Intervention paramedics referred 42 (9.6%) to falls services, compared with 17 (5.0%) of 343 participants seen by 19 control paramedics [odds ratio (OR) 2.04, 95% CI 1.12 to 3.72]. No adverse events were related to the intervention.	
• Non-significant differences between groups included subsequent emergency contacts (34.6 versus 29.1%; OR 1.27, 95% CI 0.93 to 1.72); quality of life (mean SF12 differences: MCS − 0.74, 95% CI − 45 2.83 to + 1.28; PCS − 0.13, 95% CI − 1.65 to + 1.39) and non-conveyance (42.0 versus 36.7%; OR 1.13, 95% CI 0.84 to 1.52). However, ambulance job cycle time was 8.9 min longer for intervention patients (95% CI 2.3 to 15.3).	
• Average net cost of implementing CCDS was £208 per patient with existing electronic data capture, and £308 without. Modelling estimated cost per quality-adjusted life-year at £15,000 with existing electronic data capture, and £22,200 without.	
Conclusions: Intervention paramedics referred twice as many participants to falls services with no difference in safety. CCDS is potentially cost-effective, especially with existing electronic data capture.	

### Aim

The aim of this paper is to describe paramedics’ experience of CCDS within the SAFER1 trial and to identify factors affecting its implementation and use.

## Methods

### Design

We used focus groups and interviews, at three different time points, with paramedics who had been randomly allocated to the intervention (experimental) trial arm, in order to understand the processes of change associated with the introduction of this complex healthcare intervention. It is recognised that an understanding of these mechanisms of change cannot be captured using quantitative methods alone [20–22].

We used Strong Structuration Theory (SST) [23] as the theoretical underpinning for this study, following Greenhalgh and Stones [23, 24] in incorporating a focus on the implementation of technology. SST proposes that outcomes in relation to the technology in use in an organisation are shaped by the interplay between the external structure, in this case of the NHS and the local ambulance service; the internal structure, in this case both of paramedics (their knowledge and attitude) and of the technology (its material properties and functionality); and of the actions which the paramedics as human agents take. SST also proposes that in turn outcomes can reproduce or change structures.

### Setting

We undertook the study in two UK ambulance service sites. The study was conducted in areas within these ambulance services where a falls referral pathway was available. Site one was an urban centre where we recruited paramedics from four ambulance stations and where paper clinical records were used at the outset of the trial; in site two, we recruited paramedics from nine stations across a mixed urban and rural area, and an electronic patient report form (EPRF) was in place prior to the trial, although use was variable. At site one, implementation was overseen by an operational manager, while at site two, a dedicated research paramedic was assigned to support peers in the process of adopting the intervention.

### Participants

We invited (by email, telephone call or face to face) all paramedics who had been assigned to the CCDS-use intervention arm of the SAFER 1 trial to participate in focus groups or interviews. The paramedics were either lone responders who worked from rapid response vehicles or part of double-staffed crew on emergency ambulances. Participation was voluntary, and all volunteers received a copy of the study information sheet and provided their consent to take part.

### Intervention

The intervention comprised CCDS software on a handheld computer for use by paramedics to support their assessment and decision-making about whether to take older patients who had fallen to an emergency department, or to leave them at home with referral to a community-based falls service. Paramedics recorded the patient’s past history and results of examination, via drop-down pick lists with additional prompts opening automatically as required. The CCDS covered injuries that may have been associated with the fall and co-morbidities that may have contributed to the fall (such as breathlessness or chest pain) and psycho-social needs (such as their mental state and their ability to undertake activities of daily living) plus an assessment of environmental risk. The system then generated a score indicating the recommended action, which could be logged. There was an option to print out a summary document, or to email it to a third party such as the ED. One-day training was a necessary part of the intervention, with the requirement for paramedics to gain competency in use of the CCDS.

Site one implemented CCDS alongside an EPRF and installed both on study tablet PCs; at site two, where a different EPRF was already in place, CCDS software was added to the existing system, on tablet PCs which were already in use. However, at neither site was it feasible to integrate the CCDS with the EPRF; hence, at both sites, it was up to the paramedic to decide on when to open and make use of the CCDS. During the study period, site one experienced many teething problems including loss of network signal and hardware failures which affected paramedics’ access to the CCDS.

### Data collection

Data were collected at three key time points during the trial: pre-implementation, during the trial and post-trial. Data collection was by focus group (two focus groups conducted pre-implementation; length about an hour) and by interview (*n* = 5 pre-implementation, *n* = 8 during the trial period, *n* = 9 post-trial; length 20 to 50 min). The focus groups were held at the end of initial CCDS training days at each site; because of the logistical challenges of holding focus groups with paramedic staff, telephone interviews were used to gather data from those who were not able to attend and for data collection during later stages of the study.

Semi-structured focus group topic guides and interview schedules were developed and piloted by the study team with advice from the Trial Steering Committee and local implementation teams. They were designed to explore paramedics’ attitudes towards health technology in general as well as their experience of and views on the CCDS. Data collection was conducted by researchers (two male and two female) with previous training and experience in facilitating focus groups or conducting interviews. The interviewers had no relationship with the participants prior to the commencement of the study.

Focus groups and interviews were recorded digitally and then transcribed. Transcripts were not returned to participants for comment or correction.

### Data coding and analysis

We used NVIVO software to manage the data and coded it according to the principles of ‘Framework for applied policy research’ [25], a five-stage process consisting of familiarisation, identifying a thematic framework, indexing, charting and interpretation. Two researchers took part in the coding and analysis process, to enhance objectivity.

## Results

### Sample

In total, 20 out of a possible 22 (17 male and 5 female) paramedics participated in this qualitative study. Fourteen contributed to the pre-implementation phase of data collection, eight during the trial, and nine to the post-trial data collection. Five paramedics withdrew during the course of the study due to long-term sickness or moving out of the area. The post-qualification experience as a paramedic ranged from 3 to 20 years.

### Analytical framework

**Fig. 1:**
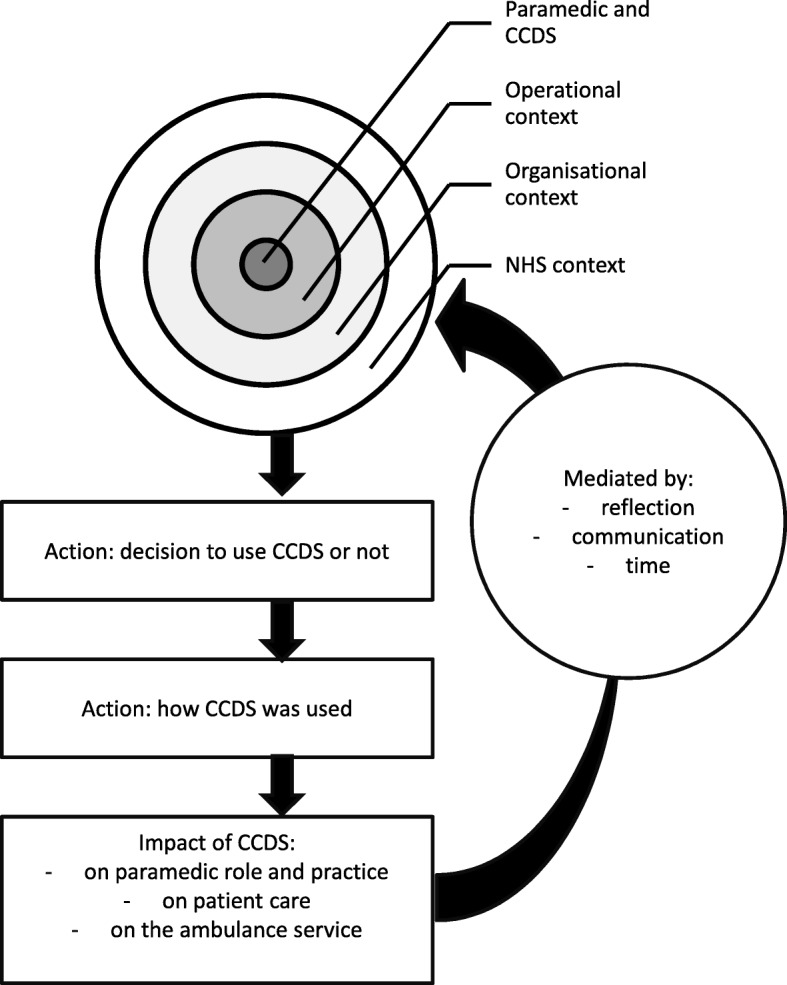
Model of CCDS implementation, adoption and impact

We developed an analytical framework consisting of codes grouped into five broad categories: personal, organisational, technical, practical and consequential (Additional file 1). Further mapping and interpretation of the data, grouping the categories and exploring the relationships between them, led to the development of a model (Fig. 1) with three domains: context, adoption and use of the CCDS by paramedics, and outcomes. We present findings in relation to these three domains, then reflect on our model in relation to Strong Structuration Theory [23, 24].

### Context

Both study sites were operating within the broad NHS context of policies to evolve the role of the paramedic, reduce unnecessary hospital attendances, meet time-based performance targets and increase the use of technology. Within this, the organisational context of each ambulance service brought aspects of structure which were formal, such as procedures and processes, and those which were more informal or implicit and related to the organisational culture. Aspects of organisational context with which paramedics interacted included the service’s existing level of digitisation, training and other processes of implementation, and perceived managerial support for paramedic decision-making.

While at site two, much of the technology infrastructure required to support the use of CCDS software was already in place prior to this study, and the software was uploaded onto the service’s existing devices alongside the EPRF system, at site one, the challenges of transitioning to new technology emerged as a barrier to implementation: the full range of hardware, software, connectivity and support systems had to be put in place, including tablets, printers, supplies of printer paper, chargers, docking stations, training, IT systems and data transfer arrangements. One paramedic at site one commented

At the very beginning when we – when we had the – the tablets, I think… that wherever we had the screens and we didn’t have the keyboards, or we had the keyboards and not the screens, and we didn’t have new printing paper. And when I said – about that, oh, all you get is a shrug of the shoulders sort of thing, like, you know…. It took months and months and months to get the right paper. (End S1 04)

In both sites, the CCDS was introduced to paramedics during formal training sessions. The majority of paramedics felt they had received enough training, a few felt they needed longer and some felt that they needed to use it in order to gain fluency. Paramedics discussed the role of the ambulance service in supporting them through the ‘transition’ to a new way of working, not just through formal training but through providing ongoing support.

Organisational culture relating to decision-making by paramedics was reported as influencing CCDS use and compliance with its recommendations, specifically around decision-making with regard to non-conveyance of patients. In site one, there were anxieties about the support which would be available from the ambulance service if a non-conveyance decision (as a result of following the CCDS guidance) led to a poor patient outcome:

This trust is pretty much, hmmm, ‘guilty until proven innocent’. So, I think a lot people want to err on the side of caution, and not leave people at home and take them in regardless of what a computer is sort of suggesting. (End S1 07)

By contrast, paramedics at site two reported that they felt there was managerial support for non-conveyance decisions, with links to alternative pathways in place.

At a more local level was paramedics’ interaction with their day to day working environment, which we have termed operational context. Paramedics at both sites reported a variety of practical challenges to using CCDS as part of their routine work, such as difficulties keeping the PC charged:

So we know roughly that the laptop won't quite last a full 12 hour shift if you're using it, even if it's, um, docked in the ambulance on charge because it's only a trickle charge, it doesn't really do a full charge. (End S2 02)

Paramedics also encountered problems with printing patient records, finding that not all vehicles were equipped with working printers, paper might be missing, or that having printers fixed to vehicles meant paramedics had to go back to their vehicles to produce print-outs and then return to their patients to give them their copy:

I kept trying to try and get it working, try and get it working, and then when it was working there was no printer paper so like it was useless anyway, so I’d gone through it and then I couldn’t print it out. (End S1 05)

In relation to the CCDS technology itself, connectivity for this web-based system was reported as a problem, particularly at site one.

The paramedics themselves and their enthusiasm, skills and motivation also formed part of the context affecting uptake. Data collected before CCDS implementation suggested that paramedics who volunteered for this study largely felt positive about the new CCDS assessment referral pathway as an opportunity to improve care for older patients who had fallen.

I think in general that paramedics, if it’s going to be beneficial for both the public and for the paramedics, they are quite open to change and they are quite eager for anything that will improve our practice … a standard approach is really needed rather than individual approaches. (Pre S1 FG2)

They seemed to be receptive to the new care model in part because of their frustrations with existing practice:

I think …the majority of us are so frustrated with the system that we just want an alternative somewhere. Pre S1 FG1

However, it was suggested that some—including colleagues who had not volunteered for the study—might be more resistant to adapting to new technology:

I think we’ve got some crew members are very resistant to change and that’s just a natural thing. They’re just worried about anything new coming in. They think – they wouldn’t bother with it. (Pre S1 FG1)

### Adoption—using CCDS

Once the CCDS had been introduced, interviews described differences between paramedics in terms of reported pattern of use: those who reported attempted use with all eligible patients; those who only used it with patients who they thought were potentially safe to be left at home; and those who never used it or discontinued use. Many paramedics felt that the software was too simple or basic to assist them with their decision-making, stating that their own clinical judgement skills and experience levels placed them in a better position than the CCDS to decide the most appropriate onward care.

I just felt that, I don’t know, some of the questions were… I don’t know, just not as advanced or a bit below paramedic level on occasions. (End S1 05)

Although the SAFER1 trial protocol required paramedics to use the CCDS on every older person who had fallen, some paramedics evolved their own approach to when they would and would not use CCDS. They reported making use of the software when they were confident that the patient would be safe to be left at home, had either decided already not to convey them or were still uncertain about whether to. However, if they had already assessed the patient and viewed them as clearly needing hospital care, they would not use the CCDS software:

If they obviously need immediate attention – like they look on the point of collapse or they’re about to die or something, then we obviously don’t use it for that, because it’s irrelevant to be honest with you, and it’s gonna get in the way of patient care. (Mid S2 03)

Decision-making was reported as also being shaped by the time of day and the situation of the patient, with the CCDS software being regarded as being a potential cause of additional delay:

And – and it was in the early hours of the morning, and it’s not a problem from our point of view because we were – we were working, [laughs] you know, but from – from – I mean the old – the – the person who had fallen had their son and his wife were there as well, and they were there because they’d been called and they were like, sort of, ‘Well, we want to go back home to bed’, you know. (Mid S2 02)

Although the CCDS was designed to be used at the point in time and place where the patient is being assessed, some paramedics reported using it retrospectively to save time, not as decision support but rather to document their assessment and care decisions.

I tended to use it after the event to be honest. I said we'll pick them up off the floor, do all our checks, decide what we're going to do then--, and then kind of go through the software. (End S2-03)

Several paramedics in the study referred to ‘grey areas’ where, having done all their assessments with a non-injured patient who has fallen, they could still be uncertain as to whether or not to convey a patient. In this situation, where paramedics were making key decisions about patient care in a context of ambiguity and uncertainty, the CCDS was also seen as a useful record of the clinical assessment and decision-making process, and confirmation that the course of action was appropriate and documented:

It sort of provided extra evidence for me to say, yes, I'm quite happy that that's the way we're going to take. (End S2 02)

And I’ve now got something recorded and written, you know, legibly, that will back my decision. (Mid S2 02)

### Outcomes

Paramedics reflected on the impact of the CCDS on their practice in relation to patient care and clinical decision-making. Several paramedics discussed how the CCDS contributed to a shift towards a greater role as independent decision-makers, without taking over from their own clinical judgement:

Clinical decision making is still my primary role, like, so it’s up to me. (End S2 04)

Many paramedics held mixed views about the CCDS, reporting benefits but also questioning the extent to which it could assist them with their decision-making.

Sometimes we have a difference of opinion between myself and the software, and I’m going to every time default to my idea on that one. And just because it – it’s quite basic software I think. (Mid S2 03)

However, aside from the CCDS’s primary purpose as decision support, paramedics cited a range of other ways in which it had an impact on patient contact and care.

- As confirmation that the course of action under consideration was appropriate:

I found that it wasn't making the decision for me, it was just agreeing with the decision that I'd already come to. (End S2 02)

The bottom line is I like this because I have got evidence to show that I have thought about what I am doing. (Pre S2 FG2)

- As a reminder and prompt to do all the necessary checks

It – it asks the question, you know, that: Is an EC- an ECG required? And it, again, you know, those sorts of things that maybe three or four o’clock in the morning, when – when you’re – when you’re not at your best is – it’s always nice to receive that kind of prompt at times. (Mid S2 08)

- As an encouragement for paramedics to reflect on their own practice:

it’s made me think more about making – about – about referring patients. (Mid S2 08)

Hmm, it got me thinking a little bit more about how we’re treating falls. (End S1 07)

One impact of the CCDS in some cases was that it may prompt paramedics to spend longer on scene with patients in order to confirm whether they are safe to leave at home. Paramedics reported that they could not predict in advance how long an assessment would take to complete, as that depended on the patient’s history, but it could be well over an hour. Against a backdrop of services under pressure, this presented anxieties for staff about whether the additional on-scene time is acceptable to managers:

I think the actual idea is good, but obviously there are times when management are getting on my case ‘cause obviously I take longer on scene than others would. (End S2 03)

The impact of the CCDS on paramedic practice was not necessarily maintained over time. In site one in particular, where there were technical problems, many paramedics reported abandoning CCDS within the first few months of the study:

It was just like I was fighting all the time to get [printer] paper or get the password or get it working, I just gave up in the end. (End S1 05)

I used it a couple of times at the beginning, and then towards the end, no. It’s just too slow. (End S1 04)

### CCDS implementation, adoption and outcomes—overview of the model

From the study findings we derived a model of implementation, adoption and impact (Fig. 1). The model includes aspects of context, which broadly correspond to the ‘structures’ of SST [16], with ‘external structures’ shaded in darker grey and ‘internal structures’ in lighter grey. The first of the external structures is the ‘NHS context’ which relates to the broad policy and resource context, including moves towards increasing provision of community-based care and reducing unnecessary hospital attendance and admissions, the shift to increasing use of technology in healthcare, and increasing the autonomy of paramedics. ‘Organisational context’ refers to the ambulance service setting and includes two key elements: firstly, the formal organisational structure, leadership and performance management processes and policies of the organisations; and secondly, the unwritten organisational culture and expectations. ‘Operational context’ refers to the day to day working environment within which the paramedic and technology function, including working with colleagues, managers and other technology. In terms of ‘internal structures’, the central box represents the paramedic as an individual practitioner and CCDS user and includes the paramedic’s attitudes, skills, motivations, expectations and adaptiveness to innovation. Alongside this human aspect sits the CCDS technology itself, its material properties and functionality.

Adoption and use of CCDS are represented on the model in the two unshaded boxes in the lower part. These correspond to the ‘action/active agency’ component of SST and describe the decision-making and actions which paramedics went through when encountering eligible patients: paramedics’ assessment of the patient in context and the decision about whether or not to use the CCDS, and, if yes, how to use it.

Finally, the impact of use of CCDS on paramedics’ role and practice, on patient care and on the ambulance service itself are noted in the dotted box at the bottom of the model. These outcomes in turn influence future use and uptake of the CCDS. We have added to this feedback loop mediating factors which our data suggested would affect how these outcomes had an influence: the reflection of paramedics on their experience, communication within the organisation and the passage of time.

## Discussion

This qualitative study examined the implementation and use of CCDS in a real-world context in two different ambulance services. While paramedics in the study were supportive of CCDS in principle, we know from the main trial that it was used with only 12% of eligible patients. Our qualitative findings help to explain why this was, and in doing so contribute to a well-documented history of partial or modified implementation of IT in health service settings [7].

Care of older people who have fallen has been identified elsewhere as being an area burdened with frustrations for paramedics and fraught with anxieties about risk and the need to ‘cover their backs’ [26–28]. Making decisions about patient care in the context of emergency ambulances is known to be complex [29, 30], so it might initially be surprising that paramedics appeared resistant to a tool designed to support their decision-making. As our qualitative data revealed, the paramedics in this study saw CCDS in conjunction with a falls referral pathway as potentially offering a timely response to non-conveyed patients as a potential solution to some of the problems they feel they encounter when deciding the best course of onward care for this patient group. However, there are many reasons why the CCDS did not become embedded into routine practice. Contextual reasons included the technical limitations of the equipment, its lack of integration with other systems as well as practical problems with it usability—all issues previously identified in studies of CCDS in other contexts [14, 15]. Paramedics had a choice about whether the effort involved in using it was worthwhile—and it seems that much of the time it was not. In their review of both electronic and non-electronic clinical decision support, Kawamoto et al. [31] identified four system features associated with interventions which were more likely to improve clinical practice. The first three were computer-based support; the provision of recommendations rather than just assessments; provision of decision support at the time and location of decision-making, all of which were found in the system we were examining. However, the fourth of Kawamoto et al.’s [31] features had the strongest association with success: automatic provision of decision support as part of the clinician workflow; in our study, by contrast, clinicians had to seek out the advice of the CCDS. This additional effort required, and interruption to the usual flow of work, may go a long way to account for low levels of use. Paramedics who did use CCDS reported choosing to use it with those patients who they had identified were ‘suitable’, rather than with all those who were ‘eligible’. For these paramedics, it was not a case of adopting it or rejecting CCDS, but developing their own approach as to how and when they used it, e.g. only with patients with whom they were uncertain about the best course of action. They appeared to have confidence in most cases in their own decision-making ability, based on past training, experience and inherent judgement, though there was a suggestion that not all paramedics would be confident in performing at the same level. Their evolving and adaptive practice in relation to CCDS offers insights to the implementing organisation with regard to how paramedics might be best placed to use new technology in practice. They identified additional benefits of the CCDS other than decision support, including providing documented evidence of the patient assessment, prompting them to do all the necessary checks and honing their skills with this patient group. Though this evolving practice might be an inevitable and useful aspect of the implementation process, non-standard working practices developed by practitioners in the field have an unknown impact on patient care [30]. Evolving and adaptive practice also highlights an inherent tension in the project of professionalising paramedic practice [32]: tools to formalise and standardise practice might be presented as part of this project, yet they can be read as challenging the autonomy of clinical decision-making which is inherent to professionalism in this context.

### Strengths and limitations

This qualitative study provides some of the first insights into how, when and why paramedics use, or attempt to use, CCDS technology and is strengthened by quantitative trial data that were considered during analysis.

This complex piece of research was carried out in the dynamic setting of the emergency services where the research agenda is not a priority; consequently, a pragmatic approach to data collection was required. Focus groups proved challenging to arrange for this clinician group which works long shifts to an irregular pattern, and semi-structured interviews were conducted instead. It is possible that richer data relating to the paramedics’ experiences and attitudes towards the CCDS and using it would have been elicited by a focus group methodology.

There were several factors to consider regarding the generalisability of these findings to other settings. Paramedics who took part in this study were volunteers (a self-selected group) rather than randomly selected; experienced paramedics were over-represented; findings are based on data from 20/22 paramedics who used CCDS as part of a pilot study with a finite time frame; and finally, that implementation problems at one site meant that CCDS usage was affected by poor usability, confusing the picture of CCDS use, and at neither site was it possible to integrate the CCDS into the EPRF workflow.

Importantly, the implementation took place in the context of an RCT, which is likely to have accentuated the issues that affect CCDS adoption by paramedics. This is both a strength and a weakness of the study, but it is important to understand what was in large part a failed implementation. The study has highlighted as a finding that leadership and managerial support were important contextual factors that were inadequately present. Given that the CCDS was introduced only as part of the trial—that is, temporarily—the managerial support that would be invested in a full IT implementation, along with the IT investment to integrate the software and address the connectivity, hardware and other issues, were unlikely to be present.

## Conclusions

The study provides useful lessons for policy makers, practitioners and researchers about the challenges to getting a new technology adopted in practice in pre-hospital care. It is a context where, previously, there has been only limited use of technology, and the introduction of CCDS presented a major shift in working practice, especially in one of the study sites.

What we have learnt from this study is that in order for an innovation such as CCDS to be used by paramedics, it is vital that systems are in place that enable its use. Implementation of new technology requires support across the organisation and is not a one-off event, but an ongoing process. Organisational and technological readiness for innovation is a factor that can impact on successful implementation of a new innovation. CCDS represents a major development in technological support for paramedics at the front line. In order for paramedics to adopt new technologies such as this, implementation needs to be supported effectively at the organisational level.

## Additional file

Additional file 1: Emerging themes and sub-themes. (DOCX 14 kb)
