# Supplementary material for: Implementation and use of computerised clinical decision support (CCDS) in emergency pre-hospital care: a qualitative study of paramedic views and experience using Strong Structuration Theory
Source: Implement Sci. 2018 Jul 4;13:91. doi: 10.1186/s13012-018-0786-x (PMC6031172; doi:10.1186/s13012-018-0786-x)
Supplement: Supplementary file 1 — Emerging themes and sub-themes. (DOCX 14 kb) [file 13012_2018_786_MOESM1_ESM.docx]

Additional file 1: Analytical codes and categories

| Category | Codes |
| --- | --- |
| Personal (paramedic) | Views on paramedic role  Attitudes to CCDS (pre and post)  IT experience and skills  Motivations and commitment  Suitability of CCDS for paramedics, patients, emergency care setting  Interpretation and ingenuity |
| Organisational (implementation) | Training  Delays post training  System functionality  Organisational support (managerial, operational, technical, colleagues)  Organisational/operational pressures and expectations  Feedback for paramedics |
| Technical (CCDS) | Functionality and fitness for purpose  Integration with ePCR  Value of clinical documentation  Paper versus computer  Audit and data retrieval  Security and confidentiality |
| Practical (adoption) | Situational considerations  Practicalities of use  Barriers and benefits to use  How and when CCDS used  Completion techniques  Future use |
| Consequential (views of impact on practice and role) | Impact on decision making  Impact on practice  Impact on patient care  Working with others  Professionalism and skills  Autonomy and risk  Demands and responsibility  Future directions |
